# Placenta Accreta Spectrum in a Patient Without Prior Cesarean Delivery: A Case of Posterior Placenta Previa

**DOI:** 10.7759/cureus.104559

**Published:** 2026-03-02

**Authors:** Vithura Kunarathnam, Mariette Sargios, Merrai Asad, Gabrielle DeGuzman, Izuka Udom-Rice

**Affiliations:** 1 Obstetrics and Gynecology, Nassau University Medical Center, East Meadow, USA; 2 Medicine, American University of the Caribbean School of Medicine, Sint Maarten, NLD

**Keywords:** atypical presentation, cesarean hysterectomy, low-risk pregnancy, magnetic resonance imaging, obstetric hemorrhage, obstetric ultrasound, placenta accreta, placenta accreta spectrum, placenta previa, posterior placenta previa

## Abstract

Placenta accreta spectrum (PAS) is a potentially fatal obstetric condition characterized by abnormal placental adherence to the myometrium. It is strongly associated with prior cesarean delivery or major uterine surgery; however, a minority of cases occur in patients without these histories. We report the case of a 34-year-old, gravida 3, para 2 (G3P2002) woman with no history of cesarean delivery and only a prior dilation and curettage, who presented at 32 weeks and 3 days of gestation with complete posterior placenta previa and imaging findings concerning for PAS. Ultrasonography revealed placental lakes and increased vascularity, while magnetic resonance imaging (MRI) demonstrated indistinct retroplacental myometrium with focal uterine bulging. The patient was counseled regarding risks, including hemorrhage, transfusion, and hysterectomy. A multidisciplinary care plan was established for cesarean hysterectomy at 34 weeks of gestation, with coordination among maternal-fetal medicine, anesthesiology, neonatology, general surgery, transfusion medicine, and intensive care services. This case highlights that PAS can present in women lacking traditional risk factors and that posterior placenta previa may pose additional diagnostic challenges, underscoring the need for heightened vigilance and multidisciplinary planning to optimize outcomes.

## Introduction

Placenta accreta spectrum (PAS) is a rare but potentially life-threatening pregnancy complication in which the placenta grows too deeply into the wall of the uterus. This abnormal attachment can prevent normal placental separation at delivery and may result in severe hemorrhage. PAS encompasses a group of disorders characterized by abnormal adherence of the placenta to the myometrium, ranging in severity from accreta to percreta [1]. The underlying problem stems from defects at the endometrial-myometrial interface, most commonly due to uterine scarring, which disrupts normal decidualization and permits deeper trophoblastic invasion into areas where the usual regulatory barriers are absent [1,2]. Importantly, the trophoblast itself is not inherently more aggressive; rather, abnormal invasion occurs because maternal physiological defenses are compromised in scarred tissue.

Rising cesarean delivery rates have contributed to a significant increase in incidence, now estimated at approximately 1 in several hundred deliveries [1,3]. Established risk factors include placenta previa, prior cesarean delivery, and other forms of uterine surgery [1,3]. Despite these well-recognized associations, a minority of PAS cases occur in women without traditional risk factors [3,4].

Posterior PAS presents additional diagnostic challenges, as characteristic imaging findings may be less apparent and the condition has been associated with increased intraoperative blood loss [3,4]. Reporting cases of PAS in low-risk populations is therefore critical to improving clinical recognition and reinforcing the need for a high index of suspicion when imaging suggests abnormal placentation. The present case describes PAS in a multiparous woman without prior cesarean delivery and with a history of dilation and curettage, highlighting that even limited uterine instrumentation may represent a clinically meaningful risk factor and that posterior placentation can further complicate prenatal diagnosis. This case underscores diagnostic limitations and highlights the importance of anticipatory, multidisciplinary management.

## Case presentation

A 34-year-old gravida 3, para 2 woman (G3P2002) at 32 weeks and 3 days of gestation was referred for evaluation of complete placenta previa with suspected PAS. The patient was asymptomatic, with no vaginal bleeding, abdominal pain, or uterine contractions. Speculum examination confirmed the absence of vaginal bleeding, and fetal heart rate tracing was reactive. She was referred following routine imaging that identified complete posterior placenta previa with features concerning for PAS. Her obstetric history included two prior uncomplicated term spontaneous vaginal deliveries: a female infant weighing 3,900 g in 2015 and a male infant weighing 3,800 g in 2020. Her medical history was notable for one prior dilation and curettage, a known potential risk factor for abnormal placentation due to disruption of the endometrial-myometrial interface, as well as group B Streptococcus bacteriuria treated with amoxicillin-clavulanate and glucose intolerance. There was no history of cesarean delivery or other major uterine surgery.

**Figure 1:**
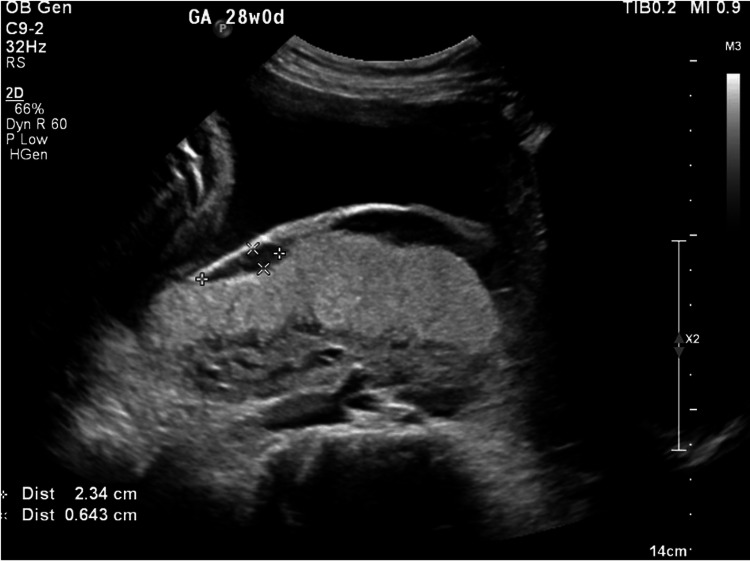
Transabdominal ultrasound demonstrating complete posterior placenta previa

**Figure 2:**
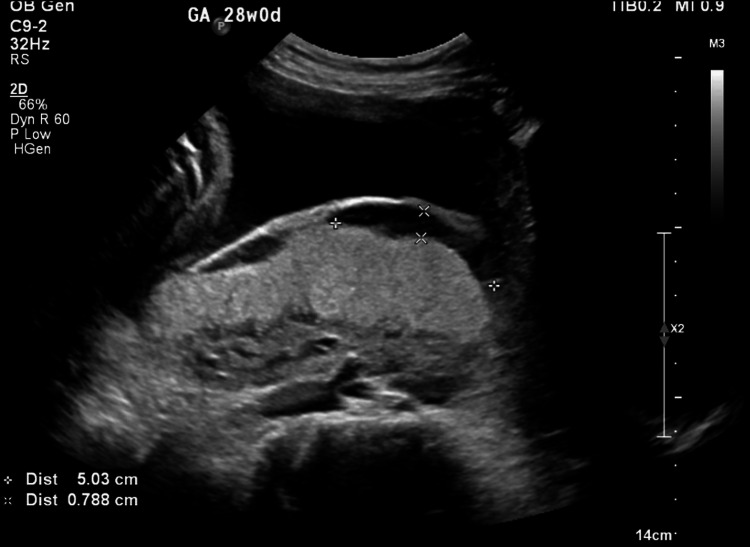
Transabdominal ultrasound demonstrating placental lakes within the posterior placenta, raising concern for abnormal placental invasion

**Figure 3:**
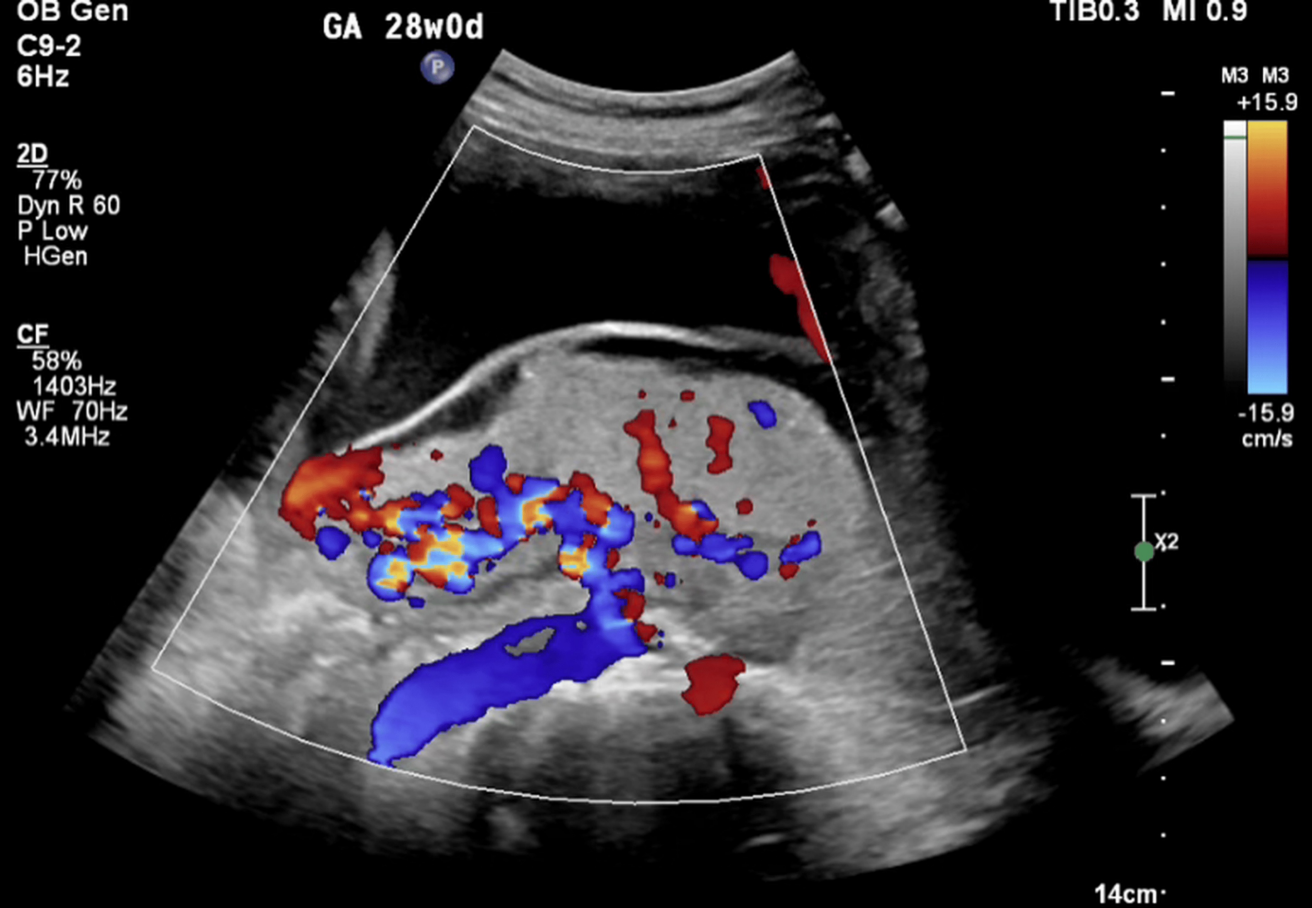
Color Doppler ultrasound demonstrating marked placental vascularity suspicious for placenta accreta spectrum

**Figure 4:**
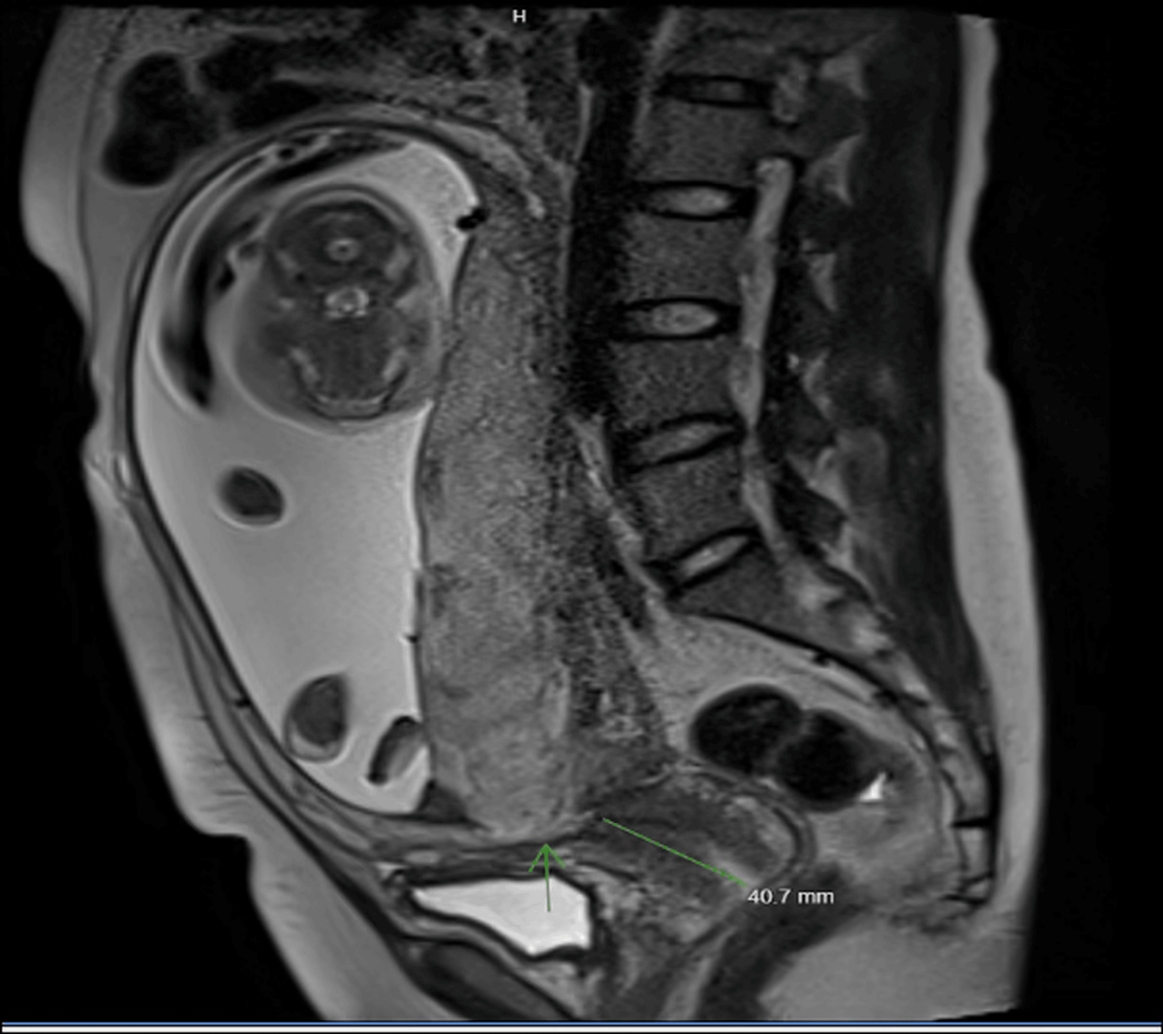
Sagittal T2-weighted pelvic MRI demonstrating indistinct retroplacental myometrium with focal uterine bulging

**Figure 5:**
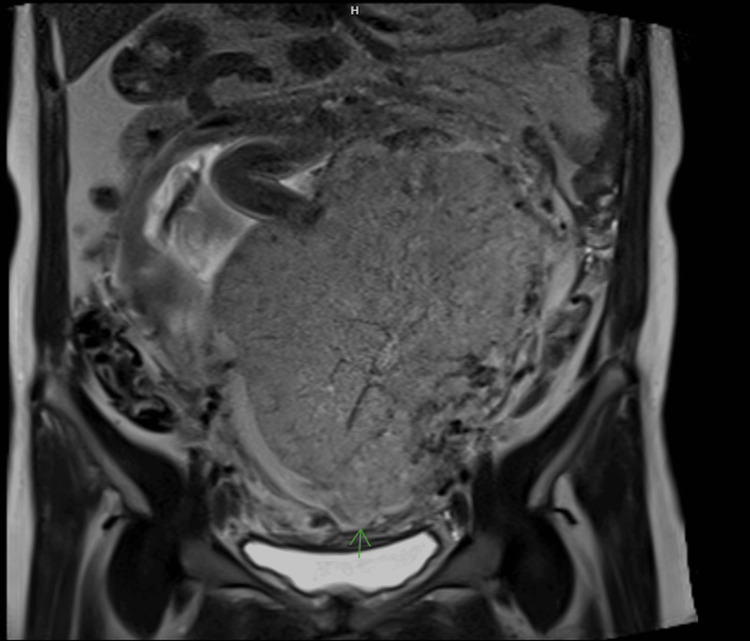
Coronal T2-weighted pelvic MRI demonstrating preservation of the bladder–uterine interface without evidence of bladder invasion

Ultrasonography revealed a complete posterior placenta previa with placental lakes and marked vascularity, raising concern for abnormal placental invasion (Figures 1-3). Magnetic resonance imaging (MRI) confirmed suspicion of PAS by demonstrating indistinct retroplacental myometrium with focal uterine bulging; however, no evidence of bladder invasion was identified (Figures 4, 5). The constellation of complete posterior placenta previa, multiple placental lakes, marked Doppler vascularity, and indistinct retroplacental myometrium with focal uterine bulging raised a strong prenatal suspicion for PAS despite the absence of prior cesarean delivery.

The patient was counseled regarding the risks of hemorrhage, transfusion, and hysterectomy. A planned delivery by cesarean hysterectomy at 34 weeks of gestation was scheduled, with hospital admission the day prior for preoperative optimization. Antenatal corticosteroids (betamethasone, Celestone) were administered to promote fetal lung maturity. Anesthesia planning included either general endotracheal anesthesia with fetal protective measures until delivery or combined neuraxial anesthesia with planned conversion to general anesthesia following delivery. General surgery and colorectal surgery were placed on standby for potential intraoperative consultation. The neonatology team discussed anticipated risks of prematurity, including respiratory distress, feeding difficulties, hyperbilirubinemia, and a likely neonatal intensive care unit stay of 2-3 weeks. The blood bank prepared four units of packed red blood cells, with platelets, fresh frozen plasma, and cryoprecipitate readily available. A postoperative intensive care unit bed was reserved for monitoring.

Hospital course

The procedure was overall uncomplicated. Intraoperative findings included a normal-appearing gravid uterus with grossly normal fallopian tubes and ovaries. The fetus was in breech presentation. A viable female neonate was delivered atraumatically with Apgar scores of 9 and 9 at 1 and 5 minutes, respectively. The posterior placenta was noted to balloon through the hysterotomy after delivery. A supracervical hysterectomy with bilateral salpingectomy was performed with the placenta left in situ. A total hysterectomy was not pursued due to significant pelvic vascular engorgement and distortion of anatomic planes, which increased the risk of injury to adjacent structures, including the bladder and ureters. A supracervical approach was therefore selected to facilitate safer hemorrhage control in this setting. The bilateral ovaries were sutured to the round ligaments to prevent postoperative torsion. Total quantified blood loss, including intraoperative and immediate postoperative periods, was 4,695 mL. Intraoperatively, the patient received four units of packed red blood cells, one unit of platelets, and one unit of fresh frozen plasma. She was transferred postoperatively to the post-anesthesia care unit and subsequently to the surgical intensive care unit for close monitoring. She was later discharged home in stable condition.

## Discussion

PAS results from abnormal placental adherence and invasion of the myometrium due to defective decidualization at the maternal-fetal interface [2]. Classically, PAS occurs at sites of prior uterine injury, most commonly following cesarean delivery, myomectomy, or other uterine surgery [1]. Posterior placentation may further complicate diagnosis, as imaging findings can be less conspicuous and have been associated with increased intraoperative blood loss [3]. In the present case, quantified blood loss reached 4,695 mL, underscoring the significant hemorrhagic risk associated with posterior PAS and supporting the need for proactive multidisciplinary planning. PAS can occasionally occur in patients without prior cesarean delivery, suggesting that additional mechanisms, such as subclinical endometrial injury, prior uterine instrumentation, multiparity, or intrinsic abnormalities of placentation, may contribute in select cases [4].

Although this patient did not have a history of cesarean delivery or major uterine surgery, she had undergone dilation and curettage. Uterine curettage may disrupt the endometrial-myometrial interface through basal layer injury, focal myometrial scarring, or the development of intrauterine adhesions. Such alterations may impair normal decidualization and predispose to abnormal placental adherence. This case underscores the importance of recognizing dilation and curettage as a potential contributor to PAS, even in patients otherwise considered low risk.

Posterior placenta previa further complicates prenatal diagnosis. Meta-analytic data demonstrate that the diagnostic accuracy of ultrasound for PAS varies by gestational age and imaging marker, with some features showing higher sensitivity and specificity in later trimesters compared to early pregnancy evaluations [5]. Characteristic ultrasound findings, including placental lakes, increased vascularity, and loss of the retroplacental clear space, may be more difficult to appreciate when the placenta is posteriorly located [3,6]. In such cases, MRI can serve as a useful adjunct, providing additional detail regarding myometrial integrity, focal uterine bulging, and the depth of placental invasion [3,6-8]. In the present case, MRI findings were instrumental in corroborating the suspicion raised on ultrasonography and guiding multidisciplinary management. Intraoperative findings and histopathologic evaluation confirmed PAS, correlating with the prenatal imaging findings of placental lakes, increased vascularity, and focal uterine bulging.

The placental bulge sign has proven valuable for identifying severe PAS, showing a sensitivity of 91.7% and specificity of 76.9% on ultrasound, and a sensitivity of 94.4% and specificity of 84.6% on MRI [9]. Recent meta-analyses have provided more detailed information on the diagnostic performance of ultrasound for PAS across different trimesters and specific sonographic markers. First-trimester ultrasound shows a sensitivity of 86% (95% CI, 78%-92%) and specificity of 63% (95% CI, 55%-70%), which is similar to second- and third-trimester performance (sensitivity 88%, specificity 92%). This suggests that routine first-trimester screening in high-risk patients may allow for earlier referral to tertiary centers [10]. Among first-trimester markers, lower uterine segment hypervascularity has the highest sensitivity at 97%, while irregularity of the uterovesical interface demonstrates the highest specificity at 99%.

Management of PAS requires a multidisciplinary approach. Current guidelines recommend planned cesarean hysterectomy at a tertiary care center, with subspecialty support and prearranged transfusion resources [1,11]. Attempted placental removal is strongly discouraged because of its association with catastrophic hemorrhage [1]. In cases where pelvic anatomy is significantly distorted by vascular engorgement, a supracervical hysterectomy may be favored over a total hysterectomy to reduce the risk of injury to adjacent structures such as the bladder and ureters while still achieving effective hemorrhage control. The present case reflected these recommendations, with coordinated involvement from maternal-fetal medicine, anesthesiology, neonatology, general surgery, transfusion medicine, and critical care. Delivery at 34 weeks of gestation balanced maternal safety with fetal maturity. Anticipatory blood bank coordination and intensive care unit availability were integral components of preoperative planning. Recent surgical frameworks emphasize standardized intraoperative staging and coordinated multidisciplinary teams to reduce maternal morbidity in PAS [11].

The present case reinforces several key clinical principles. First, PAS should be considered when imaging demonstrates abnormal vascularity, placental lakes, or disruption of the myometrial interface, even in women without prior cesarean delivery or major uterine surgery. Second, posterior placenta previa warrants heightened scrutiny, as sonographic limitations may obscure critical diagnostic features. Finally, proactive multidisciplinary planning remains central to optimizing outcomes, providing safeguards against anticipated complications such as hemorrhage, transfusion, and neonatal prematurity.

## Conclusions

PAS can develop in patients without prior cesarean delivery and may occur even in the setting of limited uterine instrumentation such as dilation and curettage. Posterior placenta previa presents unique diagnostic challenges and may obscure characteristic imaging findings. Vigilant imaging, the judicious use of MRI when ultrasonography is equivocal, and early multidisciplinary planning are essential to optimizing maternal and neonatal outcomes.
